# Detection of severe acute respiratory syndrome coronavirus 2 (SARS-CoV-2) and its first variants in fourplex real-time quantitative reverse transcription-PCR assays

**DOI:** 10.15698/mic2022.01.767

**Published:** 2021-11-25

**Authors:** Mathieu Durand, Philippe Thibault, Simon Lévesque, Ariane Brault, Alex Carignan, Louis Valiquette, Philippe Martin, Simon Labbé

**Affiliations:** 1 Plateforme RNomique et de Génomique Fonctionnelle, Université de Sherbrooke, Sherbrooke, QC, Canada.; 2 Département de Microbiologie et d'Infectiologie, Faculté de médecine et des sciences de la santé, Université de Sherbrooke, Sherbrooke, QC, Canada.; 3 Laboratoire de Microbiologie, Centre Intégré Universitaire de Santé et de Services Sociaux (CIUSSS) de l'Estrie, Centre Hospitalier Universitaire de Sherbrooke (CHUS), Sherbrooke, QC, Canada.; 4 Département de Biochimie et de Génomique Fonctionnelle, Faculté de médecine et des sciences de la santé, Université de Sherbrooke, Sherbrooke, QC, Canada.

**Keywords:** COVID-19, SARS-CoV-2, genetic variants, real-time TaqMan reverse transcription PCR assays, molecular diagnostics, locked nucleic acid (LNA)

## Abstract

The early diagnosis of severe acute respiratory syndrome coronavirus 2 (SARS-CoV-2) infections is required to identify and isolate contagious patients to prevent further transmission of SARS-CoV-2. In this study, we present a multitarget real-time TaqMan reverse transcription PCR (rRT-PCR) assay for the quantitative detection of SARS-CoV-2 and some of its circulating variants harboring mutations that give the virus a selective advantage. Seven different primer-probe sets that included probes containing locked nucleic acid (LNA) nucleotides were designed to amplify specific wild-type and mutant sequences in Orf1ab, Envelope (E), Spike (S), and Nucleocapsid (N) genes. Furthermore, a newly developed primer-probe set targeted human β_2_-microglobulin (B2M) as a highly sensitive internal control for RT efficacy. All singleplex and fourplex assays detected ≤ 14 copies/reaction of quantified synthetic RNA transcripts, with a linear amplification range of nine logarithmic orders. Primer-probe sets for detection of SARS-CoV-2 exhibited no false-positive amplifications with other common respiratory pathogens, including human coronaviruses NL63, 229E, OC43, and HKU-1. Fourplex assays were evaluated using 160 clinical samples positive for SARS-CoV-2. Results showed that SARS-CoV-2 viral RNA was detected in all samples, including viral strains harboring mutations in the Spike coding sequence that became dominant in the pandemic. Given the emergence of SARS-CoV-2 variants and their rapid spread in some populations, fourplex rRT-PCR assay containing four primer-probe sets represents a reliable approach to allow quicker detection of circulating relevant variants in a single reaction.

## INTRODUCTION

The human severe acute respiratory syndrome coronavirus 2 (SARS-CoV-2) that causes the coronavirus disease 2019 (COVID-19) started in the Chinese city of Wuhan in late 2019, and then spread around the world [1–3]. This novel coronavirus belongs to the subgroup betacoronavirus and the subgenus Sarbecovirus [3–5]. The SARS-CoV-2 genome is constituted of a positive single-stranded RNA with a genome size of nearly ~29,800 to 29,900 nucleotides in length [5]. At the beginning of the outbreak, numerous patients exhibiting an atypical viral pneumonia in Wuhan City were notified to World Health Organization [1]. Shortly thereafter, infected foreign residents of China and foreign travelers arriving in their countries of residence from international destinations, including visitors from Wuhan, contributed to the spread of the virus through person-to-person contact [4, 6, 7]. Subsequently, spreading of the virus occurred easily and rapidly from person-to-person transmission in local communities, resulting in a pandemic spread across the globe.

Although a number of vaccines against SARS-CoV-2 have been developed, they are not available to all people as their production and distribution require efficient logistic and time. Until vaccines become universally available, governments from many countries around the world strongly suggest to their citizens to maintain and sometimes enforce social distancing as they impose lockdown measures, overnight curfew and implement an obligatory quarantine in the case of international travelers flying or driving to different countries. Although rapid identification of SARS-CoV-2-infected patients is critical to trigger off their isolation, the early diagnosis of SARS-CoV-2 remains difficult for the following reasons. First, some SARS-CoV-2-infected patients exhibit no symptoms throughout the course of the infection. Second, other SARS-CoV-2-infected patients with mild symptoms could be confounded with patients who possess other kinds of atypical respiratory tract infections [8–11]. In response to the outbreak of COVID-19, numerous national public health agencies across the world have been proactive by setting up testing programs. Most of these agencies have recommended and used a nucleic-acid-based method for early detection of SARS-CoV-2. These nucleotide-based tests rely on the real-time reverse transcription PCR (rRT-PCR) that has been considered the “gold standard” approach for SARS-CoV-2 detection due to its high sensitivity for correctly identifying the viral RNA genome found in nasopharyngeal swabs and sputum samples collected from infected patients [12–14].

For daily clinical specimen testing, clinical microbiology laboratories have used different primer-probe sets for detection of SARS-CoV-2 [12, 14, 15]. Among them, the CDC N2 and Corman E (also called E Sarbeco) primer-probe sets have been identified to be particularly sensitive [15]. A typical singleplex reaction contains one set of primer pairs (forward and reverse) and a TaqMan probe that hybridizes to a specific targeted region of the viral genome. Short sequences within the nucleocapsid (N) and envelope (E) gene regions are examples of viral templates proved to be detectable with high reproducibility [12, 14]. In the same master mixture, a second set of primer pairs and TaqMan probe containing a distinct fluorophore are added to target a human gene, such as the ribonuclease P gene (RNase P also denoted RP or RPP30) to monitor nucleic acid extraction and amplification [14, 16].

Since the beginning of the COVID-19 pandemic, several studies have reported genomic sequence variations of SARS-CoV-2 isolates [17–22]. Among these genetic variations, a single nucleotide polymorphism (SNP) found at position 8,782 (in *orf1ab*; C instead of T) of SARS-CoV-2 genomic sequence is a hallmark of the L strain [23], whereas the unaltered nucleotide is found at the same position in the S strain. Several mutations have also been found in the coding sequence of the Spike protein (S-protein) [24, 25]. One critical mutation in which an adenine has been substituted by a guanine at position 23,403 in the genome of the Wuhan reference strain produces a mutant form of Spike containing D614G substitution (aspartic acid to glycine substitution at amino acid residue 614) [26]. Another critical mutation in which an adenine has been substituted by a thymine at position 23,063 generates a mutant form of Spike harboring N501Y substitution (asparagine to tyrosine substitution at amino acid residue 501) that is found in the United Kingdom (UK) B.1.1.7 strain as well as other reported variants [17]. These mutations represent few examples of the ability of SARS-CoV-2 to rapidly evolve since the beginning of the outbreak.

In this study, we report the development of novel primer-probe sets for the detection of SARS-CoV-2 variants. We use fluorogenic probes containing locked nucleic acid (LNA) for detection of SNPs in the genome of SARS-CoV-2 using the TaqMan-LNA rRT-PCR method. In addition to the new primer-probe sets, we have developed a fourplex rRT-PCR assay in which four sets of primer pairs and fluorogenic probes are contained in a single reaction master mixture. Furthermore, we have created and used a novel set of primers/probe that detects the ubiquitously expressed human β_2_-microglobulin (B2M) transcripts (instead of RNase P) as a positive control for monitoring performance of the whole procedure. These include the presence of RNAs in the collected sample, RNA extraction, reverse transcription efficacy, and real-time amplification. Thus, this multitarget rRT-PCR assay will improve our ability to narrow the differential identification of SARS-CoV-2 isolates.

## RESULTS

### Limits of detection (LoD) with SARS-CoV-2 RNA transcripts in singleplex reactions

**Figure 1:**
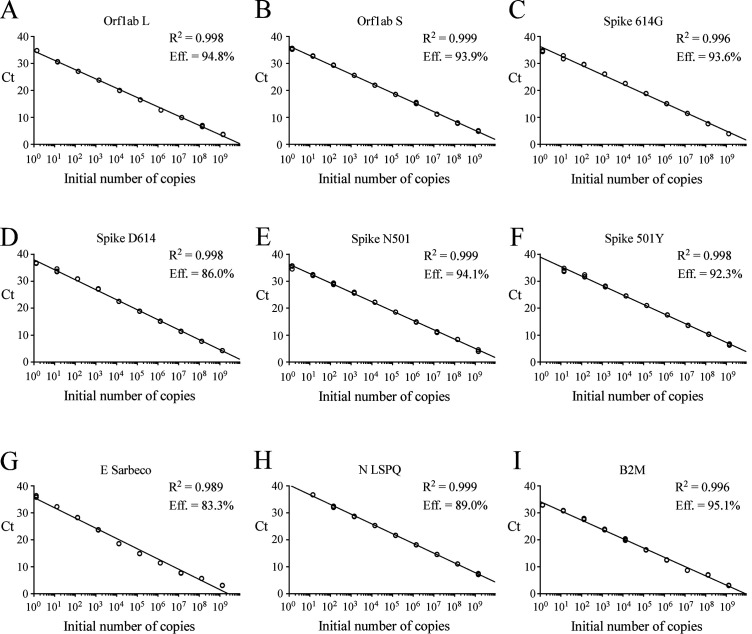
FIGURE 1: Standard curves to determine synthetic RNA copy number. (A–I) Tenfold serial dilutions ranging from 10^0^ to 10^9^ copies of Orf1ab L, Orf1ab S, Spike 614G, Spike D614, Spike N501, Spike 501Y, E Sarbeco (envelope), N LSPQ (nucleocapsid), and B2M synthetic RNA transcripts were analyzed by rRT-PCR assays. Each representative graph was generated by plotting the C_t_ values (y-axis) and the log of initial number of copies of synthetic transcripts (x-axis). Calculated linear correlation coefficients (R^2^) and percentage of amplification efficiencies are indicated for each primer-probe set. The graphs represent quantification of the results of three independent experiments.

gBlocks DNA templates containing a 5' T7 RNA polymerase promoter sequence were used to produce viral transcripts that corresponded to specific SARS-CoV-2 genomic regions (Fig. S1). These RNA molecules were used as standards for the generation of standard curves to determine the limit of detection (LoD) of each primer-probe set. Oligonucleotide primer pairs and fluorogenic probes are listed in **Table 1**. In the case of the E Sarbeco probe and primers, their nucleotide sequences were identical to those described previously [12]. The other primer pairs and fluorogenic probes were designed from the SARS-CoV-2 complete genome [1, 5]. In the cases of SARS-CoV-2 L and S strains, specific TaqMan LNA probes were designed to discriminate between the SNP found at position 8,782 (in *orf1ab*; C instead of T) of SARS-CoV-2 genomic sequence as described previously [23]. Similarly, a specific TaqMan LNA probe was synthesized to detect a guanine instead of an adenine at position 23,403 in the Wuhan reference strain [1, 5], allowing the identification of the Spike variant harboring a glycine residue instead of an aspartic acid residue at position 614 (for detection and differentiation of the 614G form versus the original D614 form) [27]. An additional mutation found in the UK strain B.1.1.7 was of interest to probe with a specific TaqMan LNA oligonucleotide to discriminate between the SNP found at position 23,063 (in *Spike*; thymine instead of adenine) where a tyrosine residue instead of an asparagine residue at position 501 was found (for detection and differentiation of the 501Y form versus the original N501 form) [28]. The sensitivity of each set of primer pair and TaqMan probe was first evaluated in singleplex reactions. Tenfold serial dilutions of viral transcript ranging from 1.3 to 1.4 x 10^9^ copies per reaction mixture were tested in triplicate by rRT-PCR assays. Results were analyzed in terms of the C_t_ value that was defined as the threshold cycle in which a target viral sequence was first detected. Samples with C_t_ values ≤ 37 were considered to be positive in comparison with background cross-reactivity of the primers and probes in non-template control (NTC) reactions. The positive C_t_ value of ≤ 37 was comparable with other reported positive C_t_ values that have been previously reported [29–33]. The highest dilution of transcript that gave a significative C_t_ value was defined as the LoD for a targeted RNA transcript. Results showed that LoD values ranged from 1.3 to 13 or 14 RNA transcript copies / reaction (**Table 2**). Linear regression curves were achieved over a 9-log dynamic range, from 1.3 to 1.3 x 10^9^ copies or 1.4 to 1.4 x 10^9^ copies per reaction for all nine probes, with calculated efficient values of 83.3% to 95.1% (**Fig. 1**).

**TABLE 1.** Oligonucleotide primers and fluorogenic probes used in real-time quantitative RT-PCR assays.

**Sets of oligonucleotides**	Nucleotide sequence*	Position in target sequence*****	**Fluorophore**
E Sarbeco F	ACAGGTACGTTAATAGTTAATAGCGT	26268-26293	
E Sarbeco R	ATATTGCAGCAGTACGCACACA	26359-26380	
E Sabo-FAM	FAM-ACACTAGCCATCCTTACTGCGCTTCG-IBFQ	28752-28776	FAM/IBFQ
Orf1ab F	TCACTCGTGACATAGCATCTAC	8711-8732	
Orf1ab R	GACTGCAGCAATCAATGGG	8817-8835	
Orf1ab strain L-LNA-TEX	TEX-CATGGTTTA**+G+*C*+C**AGCGTGGT-IBFQ	8772-8791	TEX/IBFQ
Orf1ab F	TCACTCGTGACATAGCATCTAC	8711-8732	
Orf1ab R	GACTGCAGCAATCAATGGG	8817-8835	
Orf1ab strain S-LNA-HEX	HEX-GGT**+T+T**A**+G+*T+*C**A+**G**CG-IBFQ	8775-8787**	HEX/IBFQ
Orf1ab strain S-LNA-HEX	IBFQ-CG**+C**T**+G*+A*+C**T**+A+A**ACC-HEX	reverse strand	
Spike 614G F	ACCATGTTCTTTTGGTGGTGTCA	23325-23347	
Spike 614G R	GAACCTGTAGAATAAACACGCCAAG	23456-23480	
Spike 614G LNA-HEX	HEX-TATC**+A+G+G***G***+T+G**TTAAC-IBFQ	23396-234010***	HEX/IBFQ
Spike 614G LNA-TEX	TEX-TATC+**A**+**G**+**G***G*+**T**+**G**TTAAC-IBFQ	23396-234010***	TEX/IBFQ
Spike D614 F	ACCATGTTCTTTTGGTGGTGTCA	23325-23347	
Spike D614 R	GAACCTGTAGAATAAACACGCCAAG	23456-23480	
Spike D614 LNA-FAM	FAM-TTATC**+A+G+G+*A*+T+G**TTAACT-IBFQ	23395-23411	FAM/IBFQ
Spike D614 LNA-TEX	TEX-TTATC**+A+G+G+*A*+T+G**TTAACT-IBFQ	23395-23411	TEX/IBFQ
Spike N501 F	CCGGTAGCACACCTTGTAATGG	22985-23007	
Spike N501 R	CAGTTGCTGGTGCATGTAGAAG	23111-23132	
Spike N501 LNA-FAM	FAM-ACCCACT**+*A*+A+T+G+G**TGTTG-IBFQ	23056-23072	FAM/IBFQ
Spike 501Y F	CCGGTAGCACACCTTGTAATGG	22985-23007	
Spike 501Y R	CAGTTGCTGGTGCATGTAGAAG	23111-23132	
Spike 501Y LNA-HEX	HEX-ACCCACT**+*T*+A+T+G**GTGT-IBFQ	23056-23070****	HEX/IBFQ
N LSPQ F	AACCAGAATGGAGAACGCAGTG	28351-28372	
N LSPQ R	CGGTGAACCAAGACGCAGTATTAT	28412-28435	
N LSPQ-HEX	HEX-CGATCAAAACAACGTCGGCCCCAAGGTTTAC-IBFQ	28378-28408	HEX/IBFQ
B2M F	ACTACACTGAATTCACCCCCACTGA	286-310	
B2M R	GCTGCTTACATGTCTCGATCCCA	372-394	
B2M-Cy5	Cy5-GCCTGCCGTGTGAACCATGT-IBFQ	324-343	Cy5/IBFQ
B2M-TEX	TEX-GCCTGCCGTGTGAACCATGT-IBFQ	324-343	TEX/IBFQ

*: LNA-modified nucleotides are designated with prefix ‘+' and are in bold, *e.g.* +**C** for a LNA-modified C.

**: position with variant mismatch (italic) (C instead of T at position 8782) [23].

***: position with variant mismatch (italic) (G instead of A at position 23403) [26].

****: position with variant mismatch (italic) (T instead of A at position 23063) [25].

*****: NC_045512: Reference sequence of SARS-CoV-2 isolate Wuhan-Hu-1, complete genome [1].

NM_004048 : Reference sequence of *homo sapiens* beta-2-microglobulin (B2M), mRNA.

### LoD in the case of fourplex rRT-PCR assays

**Figure 2:**
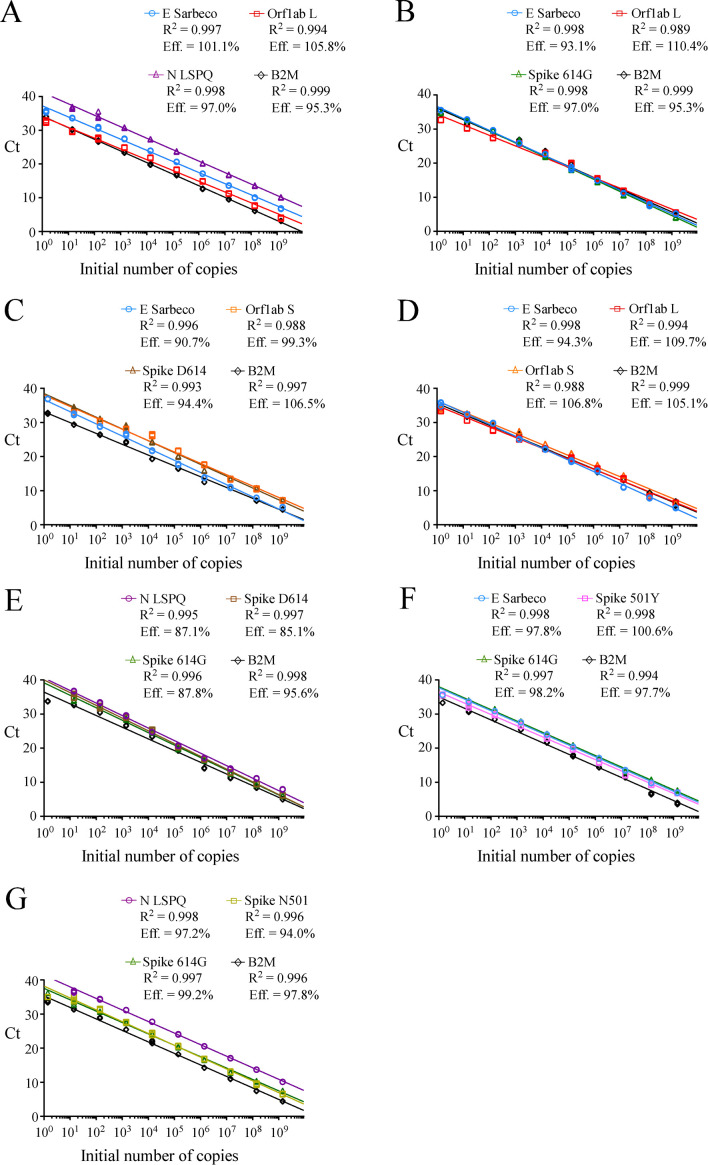
FIGURE 2: Linear regression curves to assess synthetic RNA copy number in fourplex rRT-PCR assays. (A–G) For each representative graph, four primer-probe sets within one reaction mixture were used to detect serial 10-fold dilutions of four synthetic RNA transcripts ranging from 10^0^ to 10^9^ copies per reaction. The y and x axes of each graph are the C_t_ values and the log of the input of synthetic RNA transcripts. respectively. For each fourplex reaction, R^2^ indicates calculated linear coefficients and Eff. shows percentage of amplification efficiencies for each primer-probe set that is present with three other sets of primers and probes within one reaction master mixture. Fluorogenic probe color codes are as follows: blue, E Sarbeco; red, Orf1ab L; violet, N LSPQ; black, B2M; green, Spike 614G; orange, Orf1ab S; brown, Spike D614; pink, Spike 501Y; and gold, Spike N501. The graphs represent quantification of the results of three independent experiments.

Following analytical sensitivity of the primers and probes in singleplex reactions, we sought to develop a fourplex rRT-PCR assay that contained four sets of primers and probes within one reaction master mixture. Limits of detection in the indicated fourplex assays were determined to verify whether their sensitivities were comparable to those of singleplex reactions (**Tables 2** and **3**). Fourplex reactions that were prioritized included TaqMan probes for detection of single nucleotide polymorphisms in the genome of the SARS-CoV-2 strain that had been found in patient samples [17, 26]. With the goal of identifying distinct SARS-CoV-2 virus variants, different combinations of primer-probe sets were mixed within one reaction master mixture to specifically detect viral RNA genes that have undergone genetic variations. As an example, a fourplex master mixture contained all four sets of primers and probes to determine whether a SARS-CoV-2 isolate possessed viral RNA that encoded a Spike variant harboring either an Asp^614^ or a Gly^614^ (also denoted D614G mutation) [26]. Similarly, additional fourplex master mixtures contained sets of primers and probes to determine whether a SARS-CoV-2 isolate belongs to the L or S strain [23]. Other examples of fourplex reactions included primer-probe sets to identify the SNP found in *Spike gene* at position 23,063 (thymine instead of adenine) that results in an amino acid substitution N501Y that triggers a stronger interaction of the Spike receptor-binding domain with the human cell-surface receptor angiotensin-converting enzyme 2 (ACE2) [17, 18]. This multiplex approach could facilitate tracking of SARS-CoV-2 variants that carry mutations. Serial 10-fold dilutions of SARS-CoV-2 transcripts were produced as described above and were tested using different combinations of sets of primer pairs and fluorogenic probes in one reaction mixture as described in **Table 3**. Linear amplification was performed over a 9-log dynamic range, from 1.4 to 1.4 x 10^9^ copies per tetraplex reaction for all combined probes (**Table 3**). Results showed linear regression curves that exhibited calculated efficient values of 85.1% to 110.4% (**Fig. 2**).

**TABLE 2.** Limits of detection of SARS-CoV-2-specific primers and probes using synthetic RNA transcripts in singleplex rRT-PCR assays.

**Lowest numbers of copies/reaction**	**Orf1ab L LNA-TEX**			**Orf1ab S LNA-HEX**		
	**Test 1 (Ct)**	**Test 2 (Ct)**	**Test 3 (Ct)**	**Test 1 (Ct)**	**Test 2 (Ct)**	**Test 3 (Ct)**
1.4	34.83	34.78	34.81	35.22	35.67	35.37
14	30.73	30.55	30.61	32.64	32.86	32.71
140	27.11	27.07	27.02	29.46	29.27	29.35
1.4E+03	23.85	23.81	23.78	25.64	25.63	25.55
1.4E+04	20.04	19.96	19.91	21.97	21.91	21.88
1.4E+05	16.55	16.51	16.47	18.54	18.55	18.47
1.4E+06	12.72	12.69	12.65	15.60	15.05	15.27
1.4E+07	10.03	10.00	9.92	11.22	11.19	11.11
1.4E+08	7.03	6.52	6.72	7.76	8.01	7.91
1.4E+09	3.74	3.60	3.68	4.79	5.10	4.87
NTC	Neg	Neg	Neg	Neg	Neg	Neg
**Lowest numbers of copies/reaction**	**Spike 614G LNA-HEX or TEX**			**Spike D614 LNA-FAM or TEX**		
	**Test 1 (Ct)**	**Test 2 (Ct)**	**Test 3 (Ct)**	**Test 1 (Ct)**	**Test 2 (Ct)**	**Test 3 (Ct)**
1.3	34.84	34.39	34.61	36.60	36.71	36.81
13	32.94	31.70	31.77	33.51	34.60	33.77
130	29.72	29.67	29.56	30.89	30.91	30.81
1.3E+03	26.13	26.16	26.04	27.04	27.25	27.09
1.3E+04	22.61	22.64	22.55	22.52	22.56	22.47
1.3E+05	18.82	18.91	18.85	18.88	18.95	18.81
1.3E+06	15.14	15.09	15.02	15.24	15.17	15.09
1.3E+07	11.48	11.53	11.41	11.47	11.46	11.34
1.3E+08	7.70	7.65	7.61	7.74	7.76	7.65
1.3E+09	3.89	3.91	3.97	4.30	4.31	4.22
NTC	Neg	Neg	Neg	Neg	Neg	Neg
**Lowest numbers of copies/reaction**	**Spike N501 LNA-FAM**			**Spike 501Y LNA-HEX**		
	**Test 1 (Ct)**	**Test 2 (Ct)**	**Test 3 (Ct)**	**Test 1 (Ct)**	**Test 2 (Ct)**	**Test 3 (Ct)**
1.4	35.55	35.76	34.51	nd	nd	nd
14	32.09	32.28	32.51	33.98	34.83	33.60
140	28.99	28.73	29.38	32.42	31.71	31.58
1.4E+03	25.64	25.55	25.97	27.88	27.94	28.22
1.4E+04	22.16	22.18	22.26	24.51	24.55	24.60
1.4E+05	18.58	18.54	18.54	21.01	21.04	20.95
1.4E+06	14.85	14.78	14.95	17.47	17.56	17.55
1.4E+07	11.34	10.96	10.98	13.53	13.67	13.69
1.4E+08	8.40	8.45	8.35	10.25	10.40	10.29
1.4E+09	4.02	3.92	4.60	6.23	6.79	6.41
NTC	Neg	Neg	Neg	Neg	Neg	Neg

**Lowest numbers of copies/reaction**	**E Sarbeco-FAM**		
	**Test 1 (Ct)**	**Test 2 (Ct)**	**Test 3 (Ct)**
1.3	35.79	36.46	35.96
13	32.29	32.38	32.33
130	28.33	28.21	28.27
1.3E+03	23.79	23.82	23.61
1.3E+04	18.53	18.52	18.63
1.3E+05	14.85	14.84	14.96
1.3E+06	11.39	11.38	11.44
1.3E+07	7.62	7.61	7.81
1.3E+08	5.62	5.67	5.71
1.3E+09	3.01	3.09	3.13
NTC	Neg	Neg	Neg
**Lowest numbers of copies/reaction**	**N LSPQ-HEX**		
	**Test 1 (Ct)**	**Test 2 (Ct)**	**Test 3 (Ct)**
1.4	nd	nd	nd
14	36.66	36.76	36.71
140	32.54	32.00	32.33
1.4E+03	28.80	28.76	28.58
1.4E+04	25.30	25.36	25.26
1.4E+05	21.71	21.72	21.59
1.4E+06	18.20	18.16	18.09
1.4E+07	14.65	14.64	14.51
1.4E+08	11.00	11.04	11.11
1.4E+09	7.52	7.07	7.15
NTC	Neg	Neg	Neg
**Lowest numbers of copies/reaction**	**B2M-Cy5 or TEX**		
	**Test 1 (Ct)**	**Test 2 (Ct)**	**Test 3 (Ct)**
1.3	32.90	32.84	32.97
13	30.73	30.88	30.96
130	27.98	27.49	27.64
1.3E+03	24.00	23.64	23.88
1.3E+04	20.43	19.77	20.05
1.3E+05	16.20	16.21	16.38
1.3E+06	12.52	12.44	12.61
1.3E+07	8.64	8.69	8.72
1.3E+08	7.10	6.90	6.95
1.3E+09	3.00	3.10	3.19
NTC	Neg	Neg	Neg

When we compared the sensitivity measured as the lowest C_t_ value of the TaqMan probes (Orf1ab L, Orf1ab S, Spike 614G, Spike D614, Spike N501, Spike 501Y, E Sarbeco, N LSPQ, and B2M) in singleplex reactions (**Table 2**) and fourplex reactions (**Table 3**), results showed that sensitivities of these probes between singleplex and fourplex reactions were comparable. In the cases of E Sarbeco, Orf1ab L, and B2M, their limit of detection was generally 1.3 or 1.4 copies of transcript per reaction in both singleplex and fourplex rRT-PCR assays. In the cases of Orf1ab S, Spike D614, Spike 614G, Spike N501, Spike 501Y, and N LSPQ probes, a limit of detection of 13 or 14 copies per reaction was observed with much more consistency in singleplex and fourplex assays. Taken together, all designed primer-probe sets performed comparably in singleplex and fourplex assays that reproducibly detected as few as 1.4 to 14 copies of target sequences per reaction.

**Table 3.** Sensitivities of SARS-CoV-2-specific primers and probes in fourplex rRT-PCR assays.

**Fourplex 1**												
**Probes in the fourplex**	**E Sarbeco-FAM**			**Orf1ab L LNA-TEX**			**N LSPQ-HEX**			**B2M-Cy5**		
**Lowest numbers of copies/reaction**	**Test 1 (Ct)**	**Test 2 (Ct)**	**Test 3 (Ct)**	**Test 1 (Ct)**	**Test 2 (Ct)**	**Test 3 (Ct)**	**Test 1 (Ct)**	**Test 2 (Ct)**	**Test 3 (Ct)**	**Test 1 (Ct)**	**Test 2 (Ct)**	**Test 3 (Ct)**
1.4	35.25	35.78	35.47	32.95	32.25	32.41	nd	nd	nd	33.97	34.05	33.85
14	33.53	33.70	33.61	29.48	29.71	29.56	37.03	36.39	36.68	30.12	30.31	30.04
140	30.97	30.70	30.52	27.33	27.81	27.52	35.55	33.69	34.11	26.64	27.01	26.55
1.4E+03	27.52	27.34	27.42	24.86	24.92	24.79	30.90	30.66	30.77	23.48	23.50	23.31
1.4E+04	23.93	23.97	23.88	21.89	21.75	21.78	27.28	27.30	27.42	19.85	19.97	19.71
1.4E+05	20.61	20.68	20.59	18.35	18.26	18.21	23.81	23.82	23.65	16.67	16.89	16.61
1.4E+06	17.17	17.11	17.08	14.91	14.86	14.77	20.27	20.24	20.14	12.55	12.76	12.52
1.4E+07	13.62	13.66	13.55	11.30	11.21	11.17	16.87	16.89	16.73	9.52	9.63	9.46
1.4E+08	10.09	10.01	9.94	7.70	7.58	7.63	13.69	13.58	13.51	6.07	6.10	6.01
1.4E+09	6.86	6.70	6.66	4.12	3.79	3.86	10.17	10.21	10.07	3.19	3.14	3.08
NTC	Neg	Neg	Neg	Neg	Neg	Neg	Neg	Neg	Neg	Neg	Neg	Neg
**Fourplex 2**												
**Probes in the fourplex**	**E Sarbeco-FAM**			**Orf1ab L LNA-TEX**			**Spike 614G LNA-HEX**			**B2M-Cy5**		
**Lowest numbers of copies/reaction**	**Test 1 (Ct)**	**Test 2 (Ct)**	**Test 3 (Ct)**	**Test 1 (Ct)**	**Test 2 (Ct)**	**Test 3 (Ct)**	**Test 1 (Ct)**	**Test 2 (Ct)**	**Test 3 (Ct)**	**Test 1 (Ct)**	**Test 2 (Ct)**	**Test 3 (Ct)**
1.4	35.58	35.46	35.51	32.72	32.61	32.66	34.92	34.75	34.81	34.44	34.36	34.31
14	32.67	32.85	32.72	30.20	30.32	30.24	32.57	32.83	32.66	31.68	31.40	31.48
140	29.67	29.55	29.61	27.49	27.42	27.36	29.56	29.77	29.61	29.39	29.43	29.36
1.4E+03	25.83	25.92	25.72	25.86	25.84	25.72	26.47	26.8	26.62	26.91	26.82	26.78
1.4E+04	22.53	22.70	22.61	22.84	23.05	22.75	21.85	22.20	21.94	23.60	23.61	23.52
1.4E+05	18.10	18.90	18.44	19.71	20.10	19.83	18.40	18.22	18.03	19.68	18.52	18.43
1.4E+06	14.96	14.93	14.84	15.32	15.60	15.28	14.55	14.60	14.42	15.32	15.45	15.25
1.4E+07	11.25	11.26	11.13	11.92	11.80	11.85	10.72	10.62	10.58	11.64	11.62	11.51
1.4E+08	7.99	7.54	7.41	8.23	8.20	8.16	7.74	7.80	7.69	7.90	7.91	7.86
1.4E+09	5.10	5.25	5.21	5.54	5.56	5.45	4.20	4.01	4.07	4.95	4.92	4.87
NTC	Neg	Neg	Neg	Neg	Neg	Neg	Neg	Neg	Neg	Neg	Neg	Neg
**Fourplex 3**												
**Probes in the fourplex**	**E Sarbeco-FAM**			**Orf1ab S LNA-HEX**			**Spike D614 LNA-TEX**			**B2M-Cy5**		
**Lowest numbers of copies/reaction**	**Test 1 (Ct)**	**Test 2 (Ct)**	**Test 3 (Ct)**	**Test 1 (Ct)**	**Test 2 (Ct)**	**Test 3 (Ct)**	**Test 1 (Ct)**	**Test 2 (Ct)**	**Test 3 (Ct)**	**Test 1 (Ct)**	**Test 2 (Ct)**	**Test 3 (Ct)**
1.4	36.96	36.89	36.83	nd	nd	nd	nd	nd	nd	32.67	32.85	32.51
14	32.14	32.61	32.28	32.71	32.92	32.84	33.77	34.60	34.11	29.31	29.41	29.27
140	28.78	28.91	28.72	30.46	30.38	30.27	31.08	31.14	31.01	26.47	26.49	26.31
1.4E+03	26.11	26.70	26.34	27.92	28.15	28.02	28.71	29.22	28.94	24.37	24.10	24.04
1.4E+04	21.77	21.73	21.59	25.98	26.51	26.12	24.37	24.23	24.18	19.33	19.32	19.22
1.4E+05	17.88	17.59	17.48	21.69	21.65	21.57	20.17	20.01	19.92	16.48	16.55	16.39
1.4E+06	13.77	13.83	13.65	17.64	17.61	17.49	15.91	15.93	15.82	13.85	12.54	12.45
1.4E+07	11.10	10.88	10.77	13.26	13.45	13.31	13.60	13.34	13.41	10.85	10.90	10.70
1.4E+08	7.92	7.89	7.81	10.63	10.41	10.52	10.83	10.70	10.68	7.12	7.07	7.01
1.4E+09	5.20	5.05	5.09	7.20	7.08	7.13	7.31	7.34	7.23	4.51	4.56	4.39
NTC	Neg	Neg	Neg	Neg	Neg	Neg	Neg	Neg	Neg	Neg	Neg	Neg
**Fourplex 4**												
**Probes in the fourplex**	**E Sarbeco-FAM**			**Orf1ab L LNA-TEX**			**Orf1ab S LNA-HEX**			**B2M-Cy5**		
**Lowest numbers of copies/reaction**	**Test 1 (Ct)**	**Test 2 (Ct)**	**Test 3 (Ct)**	**Test 1 (Ct)**	**Test 2 (Ct)**	**Test 3 (Ct)**	**Test 1 (Ct)**	**Test 2 (Ct)**	**Test 3 (Ct)**	**Test 1 (Ct)**	**Test 2 (Ct)**	**Test 3 (Ct)**
1.4	34.75	35.88	35.70	33.33	34.52	34.47	34.56	33.83	33.97	34.40	34.44	34.57
14	32.44	32.35	32.29	30.53	32.14	32.26	32.76	32.07	32.21	31.63	31.65	31.77
140	29.88	29.82	29.69	27.73	27.76	27.58	29.84	29.93	29.76	29.11	29.01	29.22
1.4E+03	25.17	25.13	25.01	24.97	25.41	25.12	27.48	27.50	27.39	26.52	26.54	26.62
1.4E+04	22.11	22.26	22.02	22.28	22.33	22.19	23.75	23.77	23.64	22.37	22.33	22.47
1.4E+05	18.87	18.54	18.42	19.28	19.72	19.46	20.72	21.07	20.87	18.69	19.13	19.22
1.4E+06	15.92	15.70	15.61	16.35	16.52	16.29	17.66	17.52	17.33	15.37	15.35	15.42
1.4E+07	11.07	10.97	10.88	13.45	13.60	13.32	14.56	14.48	14.39	13.07	12.99	13.21
1.4E+08	7.88	7.94	7.74	8.32	8.34	8.19	8.96	8.99	8.84	9.20	9.22	9.37
1.4E+09	4.91	5.05	4.85	6.44	6.46	6.32	6.42	6.45	6.35	6.86	5.53	6.03
NTC	Neg	Neg	Neg	Neg	Neg	Neg	Neg	Neg	Neg	Neg	Neg	Neg
**Fourplex 5**												
**Probes in the fourplex**	**N LSPQ-HEX**			**Spike D614 LNA-FAM**			**Spike 614G LNA-TEX**			**B2M-Cy5**		
**Lowest numbers of copies/reaction**	**Test 1 (Ct)**	**Test 2 (Ct)**	**Test 3 (Ct)**	**Test 1 (Ct)**	**Test 2 (Ct)**	**Test 3 (Ct)**	**Test 1 (Ct)**	**Test 2 (Ct)**	**Test 3 (Ct)**	**Test 1 (Ct)**	**Test 2 (Ct)**	**Test 3 (Ct)**
1.4	nd	nd	nd	nd	nd	nd	nd	nd	nd	33.65	33.85	33.71
14	36.68	36.81	36.72	34.88	35.94	34.76	33.95	34.15	34.03	32.68	32.71	32.59
140	33.20	33.46	33.31	31.70	32.43	32.01	31.72	31.83	31.66	30.52	30.54	30.41
1.4E+03	29.40	29.65	29.52	28.99	29.16	28.89	28.45	28.76	28.37	26.68	26.71	26.52
1.4E+04	24.67	24.75	24.69	25.11	25.46	25.22	24.68	24.88	24.55	23.45	23.48	23.33
1.4E+05	20.66	20.83	20.71	20.37	20.44	20.28	19.82	19.97	19.69	19.32	19.56	19.22
1.4E+06	17.15	17.17	17.05	16.70	16.84	16.62	16.46	16.70	16.39	14.10	14.22	14.02
1.4E+07	14.04	14.00	13.92	12.91	12.95	12.81	12.62	12.65	12.51	11.22	11.39	11.13
1.4E+08	11.15	11.21	11.08	9.58	9.57	9.41	9.29	9.31	9.19	8.41	8.47	8.32
1.4E+09	7.91	7.98	7.84	6.21	6.25	6.11	6.10	6.14	6.02	5.02	5.04	4.94
NTC	Neg	Neg	Neg	Neg	Neg	Neg	Neg	Neg	Neg	Neg	Neg	Neg
**Fourplex 6**												
**Probes in the fourplex**	**E Sarbeco-FAM**			**Spike 501Y LNA-HEX**			**Spike 614G LNA-TEX**			**B2M-Cy5**		
**Lowest numbers of copies/reaction**	**Test 1 (Ct)**	**Test 2 (Ct)**	**Test 3 (Ct)**	**Test 1 (Ct)**	**Test 2 (Ct)**	**Test 3 (Ct)**	**Test 1 (Ct)**	**Test 2 (Ct)**	**Test 3 (Ct)**	**Test 1 (Ct)**	**Test 2 (Ct)**	**Test 3 (Ct)**
1.4	nd	nd	35.64	nd	35.54	35.69	nd	nd	nd	33.31	33.33	33.29
14	33.71	33.61	33.66	32.49	31.92	32.94	33.77	34.05	33.06	30.94	30.57	30.58
140	30.84	30.85	30.90	29.73	29.70	29.95	31.51	31.11	31.14	28.41	28.48	28.49
1.4E+03	27.64	27.55	27.64	26.63	26.55	26.64	27.91	27.88	27.79	25.43	25.43	25.50
1.4E+04	24.02	23.95	23.95	22.88	22.88	22.87	24.25	24.21	24.27	21.64	21.86	21.60
1.4E+05	20.45	20.45	20.30	19.64	19.67	19.64	20.91	20.94	20.79	18.03	17.62	17.56
1.4E+06	17.12	17.09	16.98	15.88	15.96	15.72	17.19	17.26	16.94	14.39	14.39	14.31
1.4E+07	13.60	13.59	13.38	12.21	12.06	12.09	13.29	13.11	13.18	11.46	11.56	11.40
1.4E+08	9.85	9.74	9.75	9.33	9.28	9.24	10.78	10.77	10.71	6.83	6.40	6.51
1.4E+09	7.01	6.93	6.85	6.91	6.84	6.96	7.65	7.61	7.65	3.61	4.00	3.51
NTC	Neg	Neg	Neg	Neg	Neg	Neg	Neg	Neg	Neg	Neg	Neg	Neg
**Fourplex 7**												
**Probes in the fourplex**	**N LSPQ-HEX**			**Spike N501 LNA-FAM**			**Spike 614G LNA-TEX**			**B2M-Cy5**		
**Lowest numbers of copies/reaction**	**Test 1 (Ct)**	**Test 2 (Ct)**	**Test 3 (Ct)**	**Test 1 (Ct)**	**Test 2 (Ct)**	**Test 3 (Ct)**	**Test 1 (Ct)**	**Test 2 (Ct)**	**Test 3 (Ct)**	**Test 1 (Ct)**	**Test 2 (Ct)**	**Test 3 (Ct)**
1.4	nd	nd	nd	35.02	nd	nd	nd	36.2	nd	33.42	33.52	33.92
14	36.29	36.89	36.71	33.77	34.20	34.92	33.46	33.97	32.80	31.46	31.39	31.32
140	34.27	34.36	34.45	31.18	31.51	31.28	30.72	30.76	30.69	28.77	29.03	28.76
1.4E+04	27.75	27.71	27.75	23.84	24.48	24.34	24.22	24.07	24.17	21.40	21.96	22.24
1.4E+05	24.02	24.12	24.00	20.65	20.55	20.61	20.29	20.52	20.27	18.23	18.17	18.21
1.4E+06	20.56	20.58	20.51	16.73	16.90	16.81	16.60	16.61	16.53	14.21	14.27	14.29
1.4E+07	17.01	17.10	17.05	13.15	13.09	13.05	12.86	12.79	12.85	10.91	11.04	11.09
1.4E+08	13.69	13.66	13.74	9.64	9.24	9.61	10.31	10.26	10.37	7.46	7.40	7.53
1.4E+09	10.10	10.12	10.13	6.49	6.50	6.58	7.53	7.54	7.53	4.46	4.34	4.45
NTC	Neg	Neg	Neg	Neg	Neg	Neg	Neg	Neg	Neg	Neg	Neg	Neg

### Specificity of primers and probes in fourplex reactions

In the course of designing primer and probe sequences used for detection of SARS-CoV-2, we performed BLAST analyses to verify the absence of significant sequence homologies with other respiratory viruses and human genome sequences to avoid the possibility of false positive results. To test the specificity of the primers and probes that we have used in fourplex rRT-PCR reactions, we performed several assays using aliquots of the entire collection of NATtrol Respiratory Verification Panel (NATRVP) from ZeptoMetrix, which contains purified intact respiratory virus and bacteria particles that have been inactivated to make them non-infectious. These microbial particles were supplied in liquid samples containing a specialized matrix that included human cells to mimic the composition of a true clinical specimen. All SARS-CoV-2 primer and probe sets used in fourplex reactions (see **Table 3** for all combinations of four sets of primer and probes per one reaction master mixture that have been tested) showed the absence of nonspecific amplification against the ZeptoMetrix NATRVP preparation that includes 19 respiratory pathogens such as influenza A and B, different types of parainfluenza, respiratory syncytial virus A, and other coronaviruses such as NL63, 229E, OC43, and HKU-1 (**Table 4**). In contrast, the B2M primer-probe set yielded positive results for the same biological samples due to the presence of human cells that are associated with the ZeptoMetrix NATtrol Respiratory pathogen samples. As positive assays for nucleic acid detection of the 19 respiratory pathogens of the ZeptoMetrix NATRVP, we performed BioFire tests using the BioFire FilmArray 2.0 system (BioFire Diagnostics, Salt Lake City, UT). A master mixture containing all viral and bacterial particles of ZeptoMetrix was injected into the BioFire Respiratory Panel 2.1 Pouch that contained all the necessary reagents for automated nuclei acid extraction, reverse transcription, two stages PCR amplification, and detection of multiple respiratory pathogen targets in a single assay. Results showed that the FilmArray instrument in combination with BioFire Pouch reagents including multiple independent sets of primers allowed multiplex-tandem PCR detection of the microbial panel members listed in **Table 4**. Therefore, FilmArray runs validated the presence of viral and bacterial nucleic acids in the ZeptoMetrix NATRVP preparation. Taken together, the results showed that fourplex assays are specific for the SARS-CoV-2 virus target and that there is an absence of false-positive signals with other respiratory viral or bacterial pathogens.

**TABLE 4.** Specificities of fluorogenic SARS-CoV-2 probes with a number of other viruses and bacteria.

**Biofire result**			**rRT-PCR result**								
**Other viruses/bacteria**			**SARS-CoV-2**								
			**E Sarbeco**	**Orf1b strain L**	**Orf1b strain S**	**Spike D614**	**Spike 614G**	**Spike N501**	**Spike 501Y**	**N LSPQ**	**B2M**
**Viruses**	**Type and isolate (origin or year)**	**Positive (Pos) or Neg (negative)**									
Influenza A H1N1	A (New Caledonia / 1999)	Pos	Neg	Neg	Neg	Neg	Neg	Neg	Neg	Neg	Pos
Influenza A H3N2	A (Brisbane / 2007)	Pos	Neg	Neg	Neg	Neg	Neg	Neg	Neg	Neg	Pos
Influenza A H1N1	A (New York / 2009)	Pos	Neg	Neg	Neg	Neg	Neg	Neg	Neg	Neg	Pos
Influenza B	B (Florida / 2006)	Pos	Neg	Neg	Neg	Neg	Neg	Neg	Neg	Neg	Pos
Metapneumovirus	(Peru / 2003)	Pos	Neg	Neg	Neg	Neg	Neg	Neg	Neg	Neg	Pos
Respiratory Syncytial Virus (RSV)	A	Pos	Neg	Neg	Neg	Neg	Neg	Neg	Neg	Neg	Pos
Rhinovirus	1A	Pos	Neg	Neg	Neg	Neg	Neg	Neg	Neg	Neg	Pos
Parainfluenza 1	1	Pos	Neg	Neg	Neg	Neg	Neg	Neg	Neg	Neg	Pos
Parainfluenza 2	2	Pos	Neg	Neg	Neg	Neg	Neg	Neg	Neg	Neg	Pos
Parainfluenza 3	3	Pos	Neg	Neg	Neg	Neg	Neg	Neg	Neg	Neg	Pos
Parainfluenza 4	4	Pos	Neg	Neg	Neg	Neg	Neg	Neg	Neg	Neg	Pos
Adenovirus 3	3	Pos	Neg	Neg	Neg	Neg	Neg	Neg	Neg	Neg	Pos
Coronavirus NL63	NL63	Pos	Neg	Neg	Neg	Neg	Neg	Neg	Neg	Neg	Pos
Coronavirus 229E	229E	Pos	Neg	Neg	Neg	Neg	Neg	Neg	Neg	Neg	Pos
Coronavirus OC43	OC43	Pos	Neg	Neg	Neg	Neg	Neg	Neg	Neg	Neg	Pos
Coronavirus HKU-1	HKU-1	Pos	Neg	Neg	Neg	Neg	Neg	Neg	Neg	Neg	Pos
Coronavirus SARS-CoV-1	Guangdong / 2002	Pos^*^	Pos	Neg	Neg	Neg	Neg	Neg	Neg	Neg	Pos
Coronavirus MERS-CoV	Jeddah / 2012	Pos^*^	Neg	Neg	Neg	Neg	Neg	Neg	Neg	Neg	Pos
**Bacteria**											
*Mycoplasma pneumoniae*	M129	Pos	Neg	Neg	Neg	Neg	Neg	Neg	Neg	Neg	Pos
*Chlamydia pneumoniae*	CWL-029	Pos	Neg	Neg	Neg	Neg	Neg	Neg	Neg	Neg	Pos
*Bordetella pertussis*	A639	Pos	Neg	Neg	Neg	Neg	Neg	Neg	Neg	Neg	Pos

* In the two cases marked with an asterisk, results were obtained by bioinformatic analysis and rRT-PCR.

### LNA probes allow differential identification of specific SARS-CoV-2 transcripts in fourplex reactions

We tested distinct groups of differentially labeled four-color sets of probes that allow discrimination of single-nucleotide mutations found in genetic variants of SARS-CoV-2 to validate specific LNA probe-target interaction for SNPs detection in the genome of SARS-CoV-2 (**Table 5**). Results showed that the Orf1ab L probe containing a “C LNA” at the polymorphic site (position 8782 in SARS-CoV-2 sequence; **Table 1**) generated a positive fluorogenic signal that was specific for transcripts produced from the new circulating L variant strain, whereas no fluorogenic signal was detected in the presence of RNA obtained from the older S variant strain (**Table 5**; e.g. probes in fourplexes 1 and 2). In the case of the Orf1ab S probe containing a “T LNA” at the polymorphic site (**Table 1**), this probe exhibited a positive amplification signal in the presence of transcripts produced from the S variant strain and failed to detect transcripts of the L variant strain (**Table 5**; e.g. probes in fourplexes 3 and 4).

**TABLE 5.** Real-time genotyping with oligonucleotide probes containing locked nucleic acids (LNA) for detection of SARS-CoV-2 variants.

	**RT-qPCR results**			
	**Probes in the fourplex 1**			
	**E Sarbeco-FAM**	**Orf1ab L LNA-TEX**	**N LSPQ-HEX**	**B2M-Cy5**
	**Positive (Pos) or Neg (negative)**			
**RNAtemplates (140 copies/reaction)**	**Ct (values are averages of three independent assays)**			
N LSPQ	Neg	Neg	**Pos: 33.84	Neg
Orflab strain L	Neg	**Pos: 27.94	Neg	Neg
Spike 614G	Neg	Neg	Neg	Neg
B2M	Neg	Neg	Neg	**Pos: 27.32

	**RT-qPCR results**			
	**Probes in the fourplex 2**			
	**E Sarbeco-FAM**	**Orf1ab L LNA-TEX**	**Spike 614G LNAHEX**	**B2M-Cy5**
	**Positive (Pos) or Neg (negative)**			
**RNAtemplates (140 copies/reaction)**	**Ct (values are averages of three independent assays)**			
E Sarbeco	**Pos 29.15	Neg	Neg	Neg
Orflab strain S	Neg	*Neg	Neg	Neg
Spike D614	Neg	Neg	*Neg	Neg
B2M	Neg	Neg	Neg	**Pos: 28.85

	**RT-qPCR results**			
	**Probes in the fourplex 3**			
	**E Sarbeco-FAM**	**Orf1ab S LNA-TEX**	**Spike D614 LNATEX**	**B2M-Cy5**
	**Positive (Pos) or Neg (negative)**			
**RNA templates (140 copies/reaction)**	**Ct (values are averages of three independent assays)**			
E Sarbeco	**Pos: 29.28	Neg	Neg	Neg
Orf1ab strain L	Neg	*Neg	Neg	Neg
Spike 614G	Neg	Neg	*Neg	Neg
B2M	Neg	Neg	Neg	**Pos: 29.03

	**RT-qPCR results**			
	**Probes in the fourplex 4**			
	**E Sarbeco-FAM**	**Orf1ab S LNA-TEX**	**Orflab S LNAHEX**	**B2M-Cy5**
	**Positive (Pos) or Neg (negative)**			
**RNA templates (140 copies/reaction)**	**Ct (values are averages of three independent assays)**			
E Sarbeco	**Pos 29.30	Neg	Neg	Neg
Orflab strain L	Neg	**Pos: 27.91	*Neg	Neg
Spike 614G	Neg	Neg	Neg	Neg
B2M	Neg	Neg	Neg	**Pos: 28.94

	**RT-qPCR results**			
	**Probes in the fourplex 4**			
	**E Sarbeco-FAM**	**Orf1ab S LNA-TEX**	**Orflab S LNAHEX**	**B2M-Cy5**
	**Positive (Pos) or Neg (negative)**			
**RNA templates (140 copies/reaction)**	**Ct (values are averages of three independent assays)**			
E Sarbeco	**Pos: 28.97	Neg	Neg	Neg
Orflab strain S	Neg	*Neg	**Pos: 29.42	Neg
Spike D614	Neg	Neg	Neg	Neg
B2M	Neg	Neg	Neg	**Pos: 27.96

	**RT-qPCR results**			
	**Probes in the fourplex 5**			
	**N LSPQHEX**	**Spike D614 LNAFAM**	**Spike 614G LNATEX**	**B2M-Cy5**
	**Positive (Pos) or Neg (negative)**			
**RNA templates (140 copies/reaction)**	**Ct (values are averages of three independent assays)**			
E Sarbeco	Neg	Neg	Neg	Neg
Orf1ab strain L	Neg	Neg	Neg	Neg
Spike 614G	Neg	*Neg	**Pos: 31.38	Neg
B2M	Neg	Neg	Neg	**Pos: 29.25

	**RT-qPCR results**			
	**Probes in the fourplex 5**			
	**N LSPQHEX**	**Spike D614 LNAFAM**	**Spike 614G LNATEX**	**B2M-Cy5**
	**Positive (Pos) or Neg (negative)**			
**RNA templates (140 copies/reaction)**	**Ct (values are averages of three independent assays)**			
E Sarbeco	Neg	Neg	Neg	Neg
Orflab strain S	Neg	Neg	Neg	Neg
Spike D614	Neg	**Pos: 31.97	*Neg	Neg
B2M	Neg	Neg	Neg	**Pos : 27.87

	**RT-qPCR results**			
	**Probes in the fourplex 6**			
	**E Sarbeco-FAM**	**Spike 501Y LNAHEX**	**Spike 614G LNATEX**	**B2M-Cy5**
	**Positive (Pos) or Neg (negative)**			
**RNA templates (140 copies/reaction)**	**Ct (values are averages of three independent assays)**			
N LSPQ	Neg	Neg	Neg	Neg
Spike N501	Neg	*Neg	Neg	Neg
Spike 614G	Neg	Neg	**Pos: 30.11	Neg
B2M	Neg	Neg	Neg	**Pos: 28.33

	**RT-qPCR results**			
	**Probes in the fourplex 7**			
	**N LSPQHEX**	**Spike N501 LNAFAM**	**Spike 614G LNATEX**	**B2M-Cy5**
	**Positive (Pos) or Neg (negative)**			
**RNA templates (140 copies/reaction)**	**Ct (values are averages of three independent assays)**			
E Sarbeco	Neg	Neg	Neg	Neg
Spike 501Y	Neg	*Neg	Neg	Neg
Spike 614G	Neg	Neg	**Pos: 30.04	Neg
B2M	Neg	Neg	Neg	**Pos: 28.20

	**RT-qPCR results**			
	**Probes in the fourplex 8**			
	**Orf1ab L LNA-TEX**	**Spike N501 LNAFAM**	**Spike 501Y LNAHEX**	**B2M-Cy5**
	**Positive (Pos) or Neg (negative)**			
**RNA templates (140 copies/reaction)**	**Ct (values are averages of three independent assays)**			
E Sarbeco	Neg	Neg	Neg	Neg
Spike N501	Neg	**Pos : 31.24	Neg	Neg
Spike 501Y	Neg	Neg	**Pos: 29.88	Neg
B2M	Neg	Neg	Neg	**Pos: 28.04

** Pos: Positive results were ascertained according to the amplification cycle at which the fluorescence (fluorescence unit - RFU) was exponentially detected above the amplification threshold. **Pos: Ct ≤ 37.

* Neg: no signal or Ct > 37.

Neg: no signal.

Two oligonucleotide probes containing LNA residues were created for specific hybridization of target sequences containing nucleotide substitutions that confer amino acid substitution in Spike at Asp614 (also denoted D614) and Gly614 (also called 614G; **Table 1**). The LNA probe for the detection of the Wuhan reference D614 strain carried an “A LNA” at the SNP site (position 23,403 in the viral genome), whereas the fluorogenic probe specific for the detection of the circulating Spike variant (614G) contained a “G LNA” at this position (**Table 1**). In the case of the Spike D614 LNA probe, results showed that it was specific for the detection of transcripts from the Spike D614 gene and did not generate amplification signal in the presence of transcripts of the Spike 614G variant gene (**Table 5**; e.g. probes in fourplexes 3 and 5). In contrast, results showed that the Spike 614G LNA probe exhibited high specificity for detection of transcripts from the Spike 614G gene but failed to amplify transcripts from the Spike D614 coding sequence (**Table 5**; e.g. probes in fourplex 5). Similarly, experiments by fourplex rRT-PCR assays verified that the LNA probe for the detection of Spike 501Y transcripts (nucleotide change A23,063T; **Tables 1** and **2**) failed to detect the Spike N501 transcripts (**Table 5**, e.g. probes in fourplex 6). Consistently, results showed that the LNA probe for detection of the Spike N501 transcripts (nucleotide A at position 23,063; **Tables 1** and **2**) did not detect the Spike 501Y transcripts (**Table 5**, e.g. probes in fourplex 7). In contrast, results showed that the Spike N501 and Spike 501Y LNA probes exhibited high specificity for detection of transcripts from the Spike N501 and Spike 501Y coding sequences, respectively (**Table 5**, e.g. probes in fourplex 8). Taken together, the results showed that the oligonucleotide probes containing LNA residues can be used for differential identification of SARS-CoV-2 viral templates in which mutations have occurred.

### Detection of SARS-CoV-2 RNA in clinical specimens

To evaluate the use of different combinations of sets of primer pairs and fluorogenic probes in fourplex reactions for detection of SARS-CoV-2 and its variants in clinical specimens, we analyzed 160 nasopharyngeal swab samples from patients with clinical laboratory-confirmed COVID-19. Results showed that fourplex A containing probes that allow detection of double-nucleotide substitutions in the coding sequence of Spike (causing N-to-Y substitution at position 501 and D-to-G substitution at position 614) identified a total of 49 samples from patients who have been tested between February 21 and March 30, 2021 (**Table 6**). In this group of samples, the fourplex A allow the detection of all (100%) the SARS-CoV-2 501Y 614G variants when compared to data obtained by sequencing. In the case of fourplex B, sets of primer pairs and probes were combined to detect the Spike D-to-G amino acid change at position 614 whereas the original asparagine at amino acid 501 remained unchanged. A first group of 62 samples carrying the D614G substitution were found between March 19 and March 25, 2020. Furthermore, almost one year later (between February 21 and March 30, 2021), 39 samples harboring the same mutation were detected for a grand total of 101 samples (**Table 6**). Fourplex C was used to detect the SARS-CoV-2 strain L that possesses a genome encoding N501 and D614 Spike protein. Five specimens among the samples were detected harboring this genotype between March 19 and March 25, 2020 (**Table 6**). In the case of the Wuhan reference strain S in which the original amino acids at positions 501 and 614 are asparagine and aspartic acid, respectively, its detection was performed using the fourplex D. This strain was detected in five clinical specimens between March 19 and March 25, 2020 (**Table 6**). All clinical samples were confirmed to be positive with respect to their mutations or reference nucleotides by sequencing after RT-PCR followed by a second-round of PCR amplification. Taken together, the results showed that our fourplex sets of primers/probes can be effectively used in rRT-PCR assays for differential diagnosis of SARS-CoV-2 variants in clinical specimens.

**TABLE 6.** Test results using fourplex sets of primers/probes for the detection of SARS-CoV-2 variants in clinical specimens.

**SARS-CoV-2 mutations**	Number of cases among clinical specimens*	**Fourplex A**	Number of positives**	**Fourplex B**	Number of positives**	**Fourplex C**	Number of positives**	**Fourplex D**	Number of positives**
Orf1ab L with 501Y with 614G	49***	E Sarbeco-FAM	**49** (100%)	N LSPQ-HEX	**49** (100%)	Orf1ab L LNA-TEX	**49** (100%)	Orf1ab S LNA-HEX	**0** (0%)
		Spike 501Y LNA-HEX	**49** (100%)	Spike N501 LNA-FAM	**0** (0%)	Spike N501 LNA-FAM	**0** (0%)	Spike N501 LNA-FAM	**0** (0%)
		Spike 614G LNA-TEX	**49** (100%)	Spike 614G LNA-TEX	**49** (100%)	Spike 614G LNA-HEX	**49** (100%)	Spike D614 LNA-TEX	**0** (0%)
		B2M-Cy5	**49** (100%)	B2M-Cy5	**49** (100%)	B2M-Cy5	**49** (100%)	B2M-Cy5	**49** (100%)
Orf1ab L with N501 with 614G	101****	E Sarbeco-FAM	**101** (100%)	N LSPQ-HEX	**101** (100%)	Orf1ab L LNA-TEX	**101** (100%)	Orf1ab S LNA-HEX	**0** (0%)
		Spike 501Y LNA-HEX	**0** (0%)	Spike N501 LNA-FAM	**100** (99%)	Spike N501 LNA-FAM	**100** (99%)	Spike N501 LNA-FAM	**100** (99%)
		Spike 614G LNA-TEX	**101** (100%)	Spike 614G LNA-TEX	**101** (100%)	Spike 614G LNA-HEX	**101** (100%)	Spike D614 LNA-TEX	**0** (0%)
		B2M-Cy5	**101** (100%)	B2M-Cy5	**101** (100%)	B2M-Cy5	**101** (100%)	B2M-Cy5	**101** (100%)
Orf1ab L with N501 with D614	5*****	E Sarbeco-FAM	**5** (100%)	N LSPQ-HEX	**5** (100%)	Orf1ab L LNA-TEX	**5** (100%)	Orf1ab S LNA-HEX	**0** (0%)
		Spike 501Y LNA-HEX	**0** (0%)	Spike N501 LNA-FAM	**5** (100%)	Spike N501 LNA-FAM	**5** (100%)	Spike N501 LNA-FAM	5 (100%)
		Spike 614G LNA-TEX	**0** (0%)	Spike 614G LNA-TEX	**0** (0%)	Spike 614G LNA-HEX	**0** (0%)	Spike D614 LNA-TEX	**5** (100%)
		B2M-Cy5	**5** (100%)	B2M-Cy5	**5** (100%)	B2M-Cy5	**5** (100%)	B2M-Cy5	**5** (100%)
Orf1ab S with N501 with D614	5*****	E Sarbeco-FAM	**5** (100%)	N LSPQ-HEX	**5** (100%)	Orf1ab L LNA-TEX	**0** (0%)	Orf1ab S LNA-HEX	**5** (100%)
		Spike 501Y LNA-HEX	**0** (0%)	Spike N501 LNA-FAM	**5** (100%)	Spike N501 LNA-FAM	**5** (100%)	Spike N501 LNA-FAM	**5** (100%)
		Spike 614G LNA-TEX	**0** (0%)	Spike 614G LNA-TEX	**0** (0%)	Spike 614G LNA-HEX	**0** (0%)	Spike D614 LNA-TEX	**5** (100%)
		B2M-Cy5	**5** (100%)	B2M-Cy5	**5** (100%)	B2M-Cy5	**5** (100%)	B2M-Cy5	**5** (100%)
Negative clinical specimens	100****	E Sarbeco-FAM	**0** (0%)	N LSPQ-HEX	**0** (0%)	Orf1ab L LNA-TEX	**0** (0%)	Orf1ab S LNA-HEX	**0** (0%)
		Spike 501Y LNA-HEX	**0** (0%)	Spike N501 LNA-FAM	**0** (0%)	Spike N501 LNA-FAM	**0** (0%)	Spike N501 LNA-FAM	**0** (0%)
		Spike 614G LNA-TEX	**0** (0%)	Spike 614G LNA-TEX	**0** (0%)	Spike 614G LNA-HEX	**0** (0%)	Spike D614 LNA-TEX	**0** (0%)
		B2M-Cy5	**100** (100%)	B2M-Cy5	**100** (100%)	B2M-Cy5	**100** (100%)	B2M-Cy5	**100** (100%)

* All positives have been confirmed by sequencing

** Number of positive test results (% of detection).

*** Sampling period between February 21 and March 30, 2021.

**** Sampling periods between March 19 and March 25, 2020 and February 21 and March 30, 2021.

***** Sampling period between March 19 and March 25, 2020.

## DISCUSSION

The ongoing COVID-19 pandemic characterized by successive waves of infection is a driving force for optimization and constant development of SARS-CoV-2 testing assays [13, 34]. Here, we report the development of a fourplex TaqMan-LNA rRT-PCR assay that included per single reaction: four sets of primer pairs and four distinct TaqMan probes labelled with different fluorophores. Results showed that a tetraplex reaction in a single master mixture reached a detection limit of 14 viral transcript copies per reaction (**Table 3**). The amplification efficiencies of tetraplex reactions (values of 85.1% to 110.4%) were equivalent to those in the singleplex reactions (values of 83.3% and 95.1%) in which a single viral primer-probe set was used within one reaction master mixture (**Table 2**). The primer-probe set targeting a single nucleotide substitution in the Orf1ab L transcript was more sensitive than primer-probe sets targeting single nucleotide substitutions in the Orf1ab S, Spike 614G, Spike D614, Spike N501, and Spike 501Y transcripts. This set could detect transcript at levels as low as 1.4 copies per reaction in singleplex and fourplex rRT-PCR assays (**Tables 2** and **3**). In the cases of primer-probe sets targeting single nucleotide substitutions in the Orf1ab S, Spike 614G, Spike D614, Spike N501, and Spike 501Y transcripts, a detection limit of 14 transcript copies was reached when they were used in fourplex assays on three independent experiments (**Table 3**). In the case of the primer-probe set targeting the E gene (encoding the envelope membrane protein E) that is denoted E Sarbeco [12], it was more sensitive than the N LSPQ probe targeting the N gene (encoding the nucleocapsid N protein; **Tables 2** and **3**). The E Sarbeco primer-probe set could detect transcripts levels as low as 1.3 to 1.4 copies per reaction, whereas 14 transcript copies per reaction was reached with the N LSPQ primer-probe set under our experimental conditions.

During the course of studies, we have found that the human B2M gene encoding an ubiquitously expressed protein could be a positive control of choice for monitoring efficiency of RNA extraction from swab samples and subsequent reverse transcription reaction reliability. B2M is classified as one of the top gene most stably expressed with a broad range of expression in the vast majority of cell types and tissues [35]. The B2M transcript exhibits a robust half-life and its abundance is among the highest [36]. Furthermore, B2M is a top reference gene for accurate normalization in RNA expression profiles of human whole blood samples using rRT-qPCR assays [37]. To create the B2M primer-probe set and ensure that its resulting product originates from RNA/complementary DNA (cDNA) amplification and not from genomic DNA amplification, we designed the reverse primer on exon2-exon3 junction of the B2M gene. Because this exon2-exon3 boundary sequence is only present in spliced B2M mRNA and not in genomic DNA, this approach eliminated the possibility that the B2M rRT-PCR signal resulted from an amplification of genomic DNA. Results showed that the B2M primer-probe set was highly sensitive when tested in singleplex assays, exhibiting an efficiency of 95.1% (r^2^ = 0.996) and a detection limit of 1.3 transcript copies per reaction (**Fig. 1** and **Table 2**). When the B2M primer-probe set was used in fourplex assays, it remained highly sensitive with high levels of rRT-PCR efficiency (95.5% to 106.5%) and a similar detection limit (1.4 transcript copies per reaction) as compared to that observed in the case of singleplex assays (**Fig. 1** and **2**; **Tables 2** and **3**). The vast majority of COVID-19 tests are currently targeting the human RNase P gene (denoted RPP30 or h-RP) as a positive control to monitor RNA extraction and its quality to be reverse transcribed for its subsequent PCR amplification [14, 16]. However, problems with the use of one CDC-recommended h-RP primer-probe set have been reported previously [38]. One issue is that the reverse primer hybridizes in the same exon as the forward primer and the TaqMan probe, therefore allowing amplification from genomic DNA instead of exclusively from spliced mRNA [38]. In that case, a positive result with this specific primer and probe set fails to validate the quality of the clinical sample specimen with respect to its RNA content.

The use of multiplex rRT-PCR assays allowed simultaneous targeting of different regions of the SARS-CoV-2 genome in a single master mixture. Among the targeted regions, we often included the E Sarbeco probe due to its reported successful use and high sensitivity [12, 15]. In this manner, C_t_ values of the E Sarbeco probe in fourplex assays served as a point of reference to assess the significance of C_t_ values of the other tested probes. Interestingly, we have observed that utilization of multiplex SARS-CoV-2 primer/probe sets with distinct fluorophores shed light on which regions of the virus genome are more sensitive for a rRT-PCR-based SARS-CoV-2-specific identification assay. Another advantage of using a multitarget rRT-PCR method is the fact that it reduces the amount of non-enzymatic reagents, enzyme mixtures and plasticware required to analyze all the samples on a daily basis. We have found that the fourplex assay was three times cheaper as compared to the singleplex assay. This is due to the fact that each fourplex reaction contains three sets of primer pairs and three fluorogenic probes that specifically detect SARS-CoV-2 RNA genes in a single reaction master mixture (the fourth primer-probe set being used to target a human reference transcript such as B2M). In contrast, three times more reagents and three times well plates of thermal cycler(s) are required to obtain the same results using singleplex assays since the duplex approach involves three separate reaction master mixtures.

RNA viruses exhibit high mutation rates that may result in self beneficial properties by increasing their ability to infect host cells [39–41]. In the case of SARS-CoV-2, several mutations have been identified since the start of the pandemic [21, 42, 43]. For instance, two linked SNPs in the genome of SARS-CoV-2 have defined two major lineage of the virus that are designated S and L strains [23]. Another example consists of nucleotide mutations found in the viral sequence of the gene encoding Spike that result in increased virus infectivity, especially nucleotide changes resulting in amino acid changes at Asp614 (D614G) and Asn501 (N501Y) [17, 26]. Here, we have used forward and reverse primers that hybridize to each side of hot spot mutational sites found in Orf1ab (nucleotide change T8782C, generating lineage S versus L), and at different locations in Spike such as nucleotide change A23403G (resulting in Spike variation D614G) and nucleotide change A23063T (giving rise Spike variation N501Y). These pairs of primers were used in combination with wild-type and mutant-LNA probes that hybridize at the mutation site. The fourplex real-time RT-PCR assay using LNA-based TaqMan probes exploits the 5'-3' nuclease activity of Taq DNA polymerase, allowing direct detection of each of the four fluorogenic qPCR products by the uncoupling of a reporter dye from its quencher dye during qPCR. Furthermore, LNA modification of TaqMan probes increases their base pairing stability and creates a highly favorable context of duplex formation between them and their target sequences under more stringent conditions [44–47]. Therefore, these properties allow LNA-based probes to discriminate between distinct viral genomic sequences that differ by a single nucleotide at a precise hot spot mutational site. This was performed, in three different places on the genome of SARS-CoV-2 at the same time into one reaction. In this way, it is possible to detect a range of representative genotypes that could reveal the presence of different variants of SARS-CoV-2 geographically and over time. One limitation of the study is that we need to know in advance the nature of the mutation for a given SARS-CoV-2 variant that we intend to detect. However, once the nature of the mutation is known, we can design the optimal LNA-based probe to discriminate between distinct viral genomic sequences that differ by a single nucleotide at a precise mutational site.

Recently, other studies have reported multiplex rRT-PCR assays [48–49]. One study has developed triplex assays to detect SARS-CoV-2 variants of concern but only two new primer-probe sets have been designed, making it difficult to formally identify a specific variant without excluding the presence of additional mutations [48]. Another study has developed a five plex rRT-PCR test that allowed detection of three SARS-CoV-2 target RNA transcripts plus two additional control transcripts [49]. Although five primer-probe sets were designed, several results were obtained using two or three of the viral targets detected with FAM-labelled probes, which was equivalent to perform 3- or 2-plex rRT-PCR assays. One alternative to multiplex rRT-PCR approach consists of performing multi-target loop-mediated isothermal amplification (LAMP) [50]. Although real-time LAMP was specific, this method exhibited lower sensitivity compared to rRT-PCR because none of the LAMP primers were capable of detecting SARS-CoV-2 target genes down to fifty copies on patient samples [50].

When we analyzed clinical specimens, we have consistently observed that SARS-CoV-2 strains harboring the Spike gene encoding the original D614 form or belonging to the S lineage have progressively stopped to circulate in the Eastern Townships population of Quebec (Canada) through the month of March 2020 as observed in different regions throughout the world [23, 26]. After March 2020, the 614G form as well as the SARS-CoV-2 L lineage became globally dominant. Following the emergence of the SARS-CoV-2 B.1.1.7 variant in the United Kingdom on December 2020 [51, 52], within approximately two months, this variant harboring a typical N-to-Y substitution at position 501 of Spike gained prominence in clinical specimens that we have analyzed between February 21 and March 30, 2021. These above-mentioned examples illustrate the effectiveness of our method for differential diagnosis and surveillance of SARS-CoV-2 virus isolates, especially variant of concern or of high consequence as defined by the Center for Disease Control and Prevention (CDC). Furthermore, this multitarget rRT-PCR assay enables rapid follow-up of the most clinically relevant variants across the population in real-time and effectively guide the selection of viral strains that disserve further analysis by their whole-genome sequencing using next-generation sequencing methods.

## MATERIALS AND METHODS

### Primer and probe design

The Wuhan-Hu-1 genome reference sequence of SARS-CoV-2 [1, 5] as well as several other listed sequences of its genome (available in the repository of the National Center for Biotechnology Information) were aligned using BLASTn software to identify conserved nucleotide regions corresponding only to the SARS-CoV-2 viral genome and not found in other viral sequences, including SARS-CoV-1 and MERS-CoV. Subsequently, different primer-probe sets targeting distinct regions of the SARS-CoV-2 genome, especially within E, Orf1ab, and S genes, were designed using Primer Express 3.0.1 software. The N LSPQ primer-probe set was designed as described previously [53]. After validation of their exclusivity by comparing them to Virus Pathogen Resource (ViPR), Reference Viral DataBase (RVDB) and human GRCh38 genomic DNA/mRNA databases, the indicated TaqMan primer-probe sets (**Table 1**) were predicted, verified, and validated to specifically amplify SARS-CoV-2, exhibiting the absence of non-specific homologies with other respiratory viral pathogens or human-related gene sequences. The only exception was the E Sarbeco primer-probe set that was predicted to hybridize with the E gene sequence of SARS-CoV-1 in addition to the E gene of SARS-CoV-2 [12]. TaqMan probes were labeled at the 5' end with either 6-carboxyfluorescein (6-FAM), Texas Red 615 (TEX 615), hexachlorofluorescein (HEX), or Cytiva5 (Cy5) and at the 3' end with either Iowa Black FQ (IBFQ) or Black Hole Quencher 1 (BHQ1) (Integrated DNA Technologies, Coralville, Iowa).

In the case of probes containing LNA residues, they were designed for detection of SNPs in the Orf1ab and S genes of SARS-CoV-2. Essentially, we used forward and reverse primers that hybridized with each side of the target sequence and wild-type or mutant TaqMan LNA probes that hybridized at the mutation site, which was located between the forward and reverse primers. All primers and TaqMan probes were designed to avoid self-complementarity and the formation of primer-dimer by-products and hairpins. Furthermore, all primer/probe sets were validated for their compatibility in multiplex rRT-PCR assays. Standard-curve plots for calculation of rRT-PCR amplification efficiencies were performed as described previously [16, 54].

### Synthesis of RNA transcripts used as standards

gBlocks double-stranded DNA fragments encompassing the amplified region of each targeted SARS-CoV-2 region were synthesized accompanied with a T7 promoter sequence at their 5'ends (Fig. S1). A similar approach was used in the case of the DNA fragment covering the amplified region of the human B2M gene. Purified gBlocks products were used for *in vitro* T7-dependent transcription, as described previously [55]. The gBlocks DNA templates were eliminated by digestion with RNase-free DNase I for 15 min at 37°C. The RNA transcripts were purified using a MEGAclear transcription clean-up kit (Invitrogen) and were quantified spectrophotometrically at 260 nm. After RNA levels quantification, measurements of transcripts were converted to the molecule number per μl as described previously [56]. To validate calculations of viral RNA copies per reaction, we have established standard curves from 10-fold dilutions of RNA isolated from known number of copies from a prototype viral RNA preparation that was commercially available (Exact Diagnostics, Fort Worth, TX; Vircell, Granada, Spain).

### Real-time quantitative reverse transcription PCR (rRT-PCR)

Reactions were performed in a 10-μl reaction mixture including 7 μl extracted RNA-containing samples or RNA transcripts used as standards, 2.5 μl Reliance One-Step Multiplex rRT-PCR Supermix (4X concentrated) (BioRad), and 0.5 μl of forward and reverse primers (500 nM) and the indicated TaqMan or TaqMan-LNA probe (250 nM). One-step rRT-PCR amplification and detection were performed in a CFX96 Touch Real-Time PCR System (BioRad) under the following conditions. Reverse transcription of 10 min at 50°C was followed by PCR activation at 95°C for 10 min and 50 cycles of amplification (10 s at 95°C and 30 s at 60°C or for some indicated reactions at 63°C). In singleplex reaction mixtures, each reaction mixture contained a single primer pair and TaqMan or TaqMan-LNA probe. In the case of fourplex reactions, they contained all four sets of primers (500 nM per set) and probes (250 nM per probe) within a single reaction master mixture. Fluorescence measurements were monitored after each amplification round and the threshold cycle (C_t_) value for each sample was calculated by assessing the point at which fluorescence crossed the threshold line, exhibiting an increase in fluorescence above the calculated background levels. The result was considered valid if two or more of the target-specific fluorescent signals showed the C_t_ value ≤ 37 cycles and all positive and negative control reactions gave a successful and no amplification, respectively.

### BioFire FilmArray assays

The collection of respiratory viral and bacterial pathogens was obtained from ZeptoMetrix (cat #NATRVP-IDI). The collection is called NATtrol Respiratory Verification Panel (NATRVP) and contains 20 vials x 0.6 ml, each containing viral and bacterial targets. A master mixture that was constituted of an aliquot of each pathogen of NATRVP was injected into the BioFire Respiratory Panel 2.1 (RP2.1) Pouch in accordance with the manufacturer's instructions for analyze using the BioFire FilmArray 2.0 System (BioFire Diagnostics) as described previously [57]. The loaded RP2.1 Pouch inserted into the FilmArray instrument contained all necessary reagents for automated nucleic acid extraction, reverse transcription, and two consecutive multiplex PCR amplification runs. Furthermore, the FilmArray instrument has the potential to undergo gene target melt curve analysis with each target in a valid run reported as detected or not detected.

### Clinical specimens

Deidentified nasopharyngeal swabs from infected individuals were obtained from the *Laboratoire de Microbiologie du Centre Intégré Universitaire de Santé et de Services Sociaux de l'Estrie, Centre Hospitalier Universitaire de Sherbrooke*. The thermal inactivation of SARS-CoV-2 was performed using 20 μl of nasopharyngeal swab diluent that was mixed with sterile water (20 μl). The sample preparation was heated at 90°C for 2 min and then chilled at 4°C for 3 min. Following this step, 5 μl of heat-inactivated SARS-CoV-2-containing sample was analyzed by direct one-step rRT-PCR as described above in the previous section dedicated to the rRT-PCR method. Positive fourplex rRT-PCR results were confirmed by retesting the samples in singleplex rRT-PCR reactions from which DNA products were separately analyzed by DNA sequencing to confirm identity of each DNA sequence and discriminate between two (or more) viral genomic sequences that differ by a single nucleotide. Aside thermal preparation, we have also performed simultaneous RNA extractions using the MagNA Pure Compact system (Roche). Nucleic acid extracts gave the same results in the case of positive samples of SARS-CoV-2 among the specimens assessed.

The authority that provided ethics approval is the *Centre Intégré Universitaire de Santé et de Services Sociaux (CIUSSS) de l'Estrie-CHUS*. The CIUSSSE-CHUS institutional review board approved this study #2021-4223. The requirement for informed consent was waived because this was a retrospective study that used unmarked and deidentified leftover samples.

## AUTHOR CONTRIBUTIONS

M.D. conceptualized, performed and analyzed rRT-PCR data. P.T. conceptualized the design of primers and TaqMan-LNA probes as well as carrying numerous bioinformatic analyses. S. Lévesque conceptualized and performed BioFire FilmArray assays. A.B. acquired and analyzed the data. A.C, L.V., and P.M. conceptualized the experimental work and analyzed data. S. Labbé conceptualized the experimental work and wrote the manuscript. The authors reviewed the results and approved the final version of the manuscript.

## SUPPLEMENTAL MATERIAL

Click here for supplemental data file.

All supplemental data for this article are available online at https://www.microbialcell.com/researcharticles/2021a-durand-microbial-cell/.

## Abbreviations:

B2M: – β_2_ microglobulin,
COVID-19: – coronavirus disease 2019,
LAMP: – loop-mediated isothermal amplification,
LNA: – locked nucleic acid,
LoD: – limit of detection,
rRT-PCR: – real-time reverse transcription PCR,
SARS-CoV-2: – severe acute respiratory syndrome coronavirus 2,
SNP: – single nucleotide polymorphism.
