# Supplementary material for: Detection of severe acute respiratory syndrome coronavirus 2 (SARS-CoV-2) and its first variants in fourplex real-time quantitative reverse transcription-PCR assays
Source: Microb Cell. 2021 Nov 25;9(1):1–20. doi: 10.15698/mic2022.01.767 (PMC8717086; doi:10.15698/mic2022.01.767)
Supplement: Supplementary file 1 [file mic-09-001-s01.pdf]

Detection of severe acute respiratory syndrome coronavirus 2 (SARS-CoV-2) and its first variants in fourplex real-time quantitative reverse transcription-PCR assays.

Mathieu Durand[2], Philippe Thibault[2], Simon Lévesque[3,4], Ariane Brault[1], Alex Carignan[3], Louis Valiquette[3], Philippe Martin[3], and Simon Labbé[1]*

[1]Département de Biochimie et de Génomique Fonctionnelle, Faculté de médecine et des sciences de la santé, Université de Sherbrooke, Sherbrooke, QC, J1E 4K8, Canada.
[2]Plateforme RNomique et de Génomique Fonctionnelle, Université de Sherbrooke, Sherbrooke, QC, Canada.
[3]Département de Microbiologie et d'Infectiologie, Faculté de médecine et des sciences de la santé, Université de Sherbrooke, Sherbrooke, QC, J1E 4K8, Canada.
[4]Laboratoire de Microbiologie, Centre Intégré Universitaire de Santé et de Services Sociaux (CIUSSS) de l'Estrie, Centre Hospitalier Universitaire de Sherbrooke (CHUS), Sherbrooke, QC, J1H 5N4, Canada.

*Corresponding author : Simon Labbé

E-mail: Simon.Labbe@USherbrooke.ca

TABLE OF CONTENT : Supplemental Figure 1.

Legend

Fig. S1. *Sequences of the synthetic oligomers used to create the indicated gBlocks*. Each targeted SARS-CoV-2 region from the Orf1ab, Spike, E (envelope), and N (nucleocapsid) genes (according to the Wuhan-Hu-1 genome reference sequence and mutant derivatives) were designed accompanied with a T7 promoter sequence (bold) at their 5'ends.

Supplemental Figure 1. Sets of oligonucleotide primers

Orf1ab forward and reverse (strain L)
5'-**TAATACGACTCACTATAGG**ATGGTGGTGTCACTCGTGACATAGCATCTACAGATACTTGTTTTGCTAACAAACATGCTGATTTTGACACATGGTTTAGCC
AGCGTGGTGGTAGTTATACTAATGACAAAGCTTGCCCATTGATTGCTGCAGTCATAACA-3'
5'-TGTTATGACTGCAGCAATCAATGGGCAAGCTTTGTCATTAGTATAACTACCACCACGCTGGCTAAACCATGTGTCAAAATCAGCATGTTTGTTAGCAAAA
CAAGTATCTGTAGATGCTATGTCACGAGTGACACCACCATCCTATAGTGAGTCGTATTA-3'

Orf1ab forward and reverse (strain S)
5'-**TAATACGACTCACTATAGG**ATGGTGGTGTCACTCGTGACATAGCATCTACAGATACTTGTTTTGCTAACAAACATGCTGATTTTGACACATGGTTTAGTC
AGCGTGGTGGTAGTTATACTAATGACAAAGCTTGCCCATTGATTGCTGCAGTCATAACA-3'
5'-TGTTATGACTGCAGCAATCAATGGGCAAGCTTTGTCATTAGTATAACTACCACCACGCTGACTAAACCATGTGTCAAAATCAGCATGTTTGTTAGCAAAA
CAAGTATCTGTAGATGCTATGTCACGAGTGACACCACCATCCTATAGTGAGTCGTATTA-3'

Spike D614 forward and reverse
5'-**TAATACGACTCACTATAGG**CTTGACATTACACCATGTTCTTTTGGTGGTGTCAGTGTTATAACACCAGGAACAAATACTTCTAACCAGGTTGCTGTTCTT
TATCAGGATGTTAACTGCACAGAAGTCCCTGTTGCTATTCATGCAGATCAACTTACTCCTACTTGGCGTGTTTATTCTACAGGTTCTAATGTTT-3'
5'-AAACATTAGAACCTGTAGAATAAACACGCCAAGTAGGAGTAAGTTGATCTGCATGAATAGCAACAGGGACTTCTGTGCAGTTAACATCCTGATAAAGAAC
AGCAACCTGGTTAGAAGTATTTGTTCCTGGTGTTATAACACTGACACCACCAAAAGAACATGGTGTAATGTCAAGCCTATAGTGAGTCGTATTA-3'

Spike 614G forward and reverse
5'-**TAATACGACTCACTATAGG**CTTGACATTACACCATGTTCTTTTGGTGGTGTCAGTGTTATAACACCAGGAACAAATACTTCTAACCAGGTTGCTGTTCTT
TATCAGGGTGTTAACTGCACAGAAGTCCCTGTTGCTATTCATGCAGATCAACTTACTCCTACTTGGCGTGTTTATTCTACAGGTTCTAATGTTT-3'
5'-AAACATTAGAACCTGTAGAATAAACACGCCAAGTAGGAGTAAGTTGATCTGCATGAATAGCAACAGGGACTTCTGTGCAGTTAACACCCTGATAAAGAAC
AGCAACCTGGTTAGAAGTATTTGTTCCTGGTGTTATAACACTGACACCACCAAAAGAACATGGTGTAATGTCAAGCCTATAGTGAGTCGTATTA-3'

Spike N501 forward and reverse
5'-**TAATACGACTCACTATAGG**AACCTTTTGAGAGAGATATTTCAACTGAAATCTATCAGGCCGGTAGCACACCTTGTAATGGTGTTGAAGGTTTTAATTGTT
ACTTTCCTTTACAATCATATGGTTTCCAACCCACTAATGGTGTTGGTTACCAACCATACAGAGTAGTAGTACTTTCTTTTGAACTTCTACATGCACCAGCAAC
TGTTTGTGGACC
5'-GGTCCACAAACAGTTGCTGGTGCATGTAGAAGTTCAAAAGAAAGTACTACTACTCTGTATGGTTGGTAACCAACACCATTAGTGGGTTGGAAACCATATG
ATTGTAAAGGAAAGTAACAATTAAAACCTTCAACACCATTACAAGGTGTGCTACCGGCCTGATAGATTTCAGTTGAAATATCTCTCTCAAAAGGTTCCTATAG
TGAGTCGTATTA-3'

Spike 501Y forward and reverse
5'-**TAATACGACTCACTATAGG**AACCTTTTGAGAGAGATATTTCAACTGAAATCTATCAGGCCGGTAGCACACCTTGTAATGGTGTTGAAGGTTTTAATTGTT
ACTTTCCTTTACAATCATATGGTTTCCAACCCACTTATGGTGTTGGTTACCAACCATACAGAGTAGTAGTACTTTCTTTTGAACTTCTACATGCACCAGCAAC
TGTTTGTGGACC
5'-GGTCCACAAACAGTTGCTGGTGCATGTAGAAGTTCAAAAGAAAGTACTACTACTCTGTATGGTTGGTAACCAACACCATAAGTGGGTTGGAAACCATATG
ATTGTAAAGGAAAGTAACAATTAAAACCTTCAACACCATTACAAGGTGTGCTACCGGCCTGATAGATTTCAGTTGAAATATCTCTCTCAAAAGGTTCCTATAG
TGAGTCGTATTA-3'

E Sarbeco forward and reverse
5'-**TAATACGACTCACTATAGG**ATGTACTCATTCGTTTCGGAAGAGACAGGTACGTTAATAGTTAATAGCGTACTTCTTTTTCTTGCTTTCGTGGTATTCTTG
CTAGTTACACTAGCCATCCTTACTGCGCTTCGATTGTGTGCGTACTGCTGCAATATTGTTAACGTGAGTCTTGTAAAACCTTCTTTTTACGTTTACTCTCGTG
TTAAAAATCTGAATTCTTCTAGAGTTCCTGATCTTCTGGTCTAA-3'
5'-TTAGACCAGAAGATCAGGAACTCTAGAAGAATTCAGATTTTTAACACGAGAGTAAACGTAAAAAGAAGGTTTTACAAGACTCACGTTAACAATATTGCAG
CAGTACGCACACAATCGAAGCGCAGTAAGGATGGCTAGTGTAACTAGCAAGAATACCACGAAAGCAAGAAAAAGAAGTACGCTATTAACTATTAACGTACCTG
TCTCTTCCGAAACGAATGAGTACATCCTATAGTGAGTCGTATTA-3'

N LSPQ forward and reverse
5'-**TAATACGACTCACTATAGG**GTTTGGTGGACCCTCAGATTCAACTGGCAGTAACCAGAATGGAGAACGCAGTGGGGCGCGATCAAAACAACGTCGGCCCCA
AGGTTTACCCAATAATACTGCGTCTTGGTTCACCGCTCTCACTCAACATGGCAAGGAAGACCTTAAATT-3'
5'-AATTTAAGGTCTTCCTTGCCATGTTGAGTGAGAGCGGTGAACCAAGACGCAGTATTATTGGGTAAACCTTGGGGCCGACGTTGTTTTGATCGCGCCCCAC
TGCGTTCTCCATTCTGGTTACTGCCAGTTGAATCTGAGGGTCCACCAAACCCTATAGTGAGTCGTATTA-3'

B2M exons 2-3 forward and reverse
5'-**TAATACGACTCACTATAGG**GTACTCCAAAGATTCAGGTTTACTCACGTCATCCAGCAGAGAATGGAAAGTCAAATTTCCTGAATTGCTATGTGTCTGGGT
TTCATCCATCCGACATTGAAGTTGACTTACTGAAGAATGGAGAGAGAATTGAAAAAGTGGAGCATTCAGACTTGTCTTTCAGCAAGGACTGGTCTTTCTATCT
CTTGTACTACACTGAATTCACCCCCACTGAAAAAGATGAGTATGCCTGCCGTGTGAACCATGTGACTTTGTCACAGCCCAAGATAGTTAAGTGGGATCGAGAC
ATGTAAGCAGCATCATGGAG-3'
5'-CTCCATGATGCTGCTTACATGTCTCGATCCCACTTAACTATCTTGGGCTGTGACAAAGTCACATGGTTCACACGGCAGGCATACTCATCTTTTTCAGTGG
GGGTGAATTCAGTGTAGTACAAGAGATAGAAAGACCAGTCCTTGCTGAAAGACAAGTCTGAATGCTCCACTTTTTCAATTCTCTCTCCATTCTTCAGTAAGTC
AACTTCAATGTCGGATGGATGAAACCCAGACACATAGCAATTCAGGAAATTTGACTTTCCATTCTCTGCTGGATGACGTGAGTAAACCTGAATCTTTGGAGTA
CCCTATAGTGAGTCGTATTA-3'

Bold sequences indicate T7 promoter sequence that was used for RNA synthesis

Fig. S1 Durand et al.
